# Changes in intestinal morphology, number of mucus-producing cells and expression of coronavirus receptors APN, DPP4, ACE2 and TMPRSS2 in pigs with aging

**DOI:** 10.1186/s13567-023-01169-7

**Published:** 2023-04-13

**Authors:** Waqar Saleem, Xiaolei Ren, Wim Van Den Broeck, Hans Nauwynck

**Affiliations:** 1 Laboratory of Virology, Department of Translational Physiology, Infectiology and Public Health, Faculty of Veterinary Medicine, Ghent University, 9820 Merelbeke, Belgium; 2 Department of Morphology, Imaging, Orthopedics, Rehabilitation and Nutrition, Faculty of Veterinary Medicine, Ghent University, 9820 Merelbeke, Belgium

**Keywords:** Porcine intestines, morphometry, mucus-producing cells, expression of coronavirus receptors

## Abstract

Porcine enteric viral infections cause high morbidity and mortality in young piglets (<3 weeks). Later, these rates decrease with age. This age-dependent infectivity remains largely unexplored. This study investigated the changes in intestinal morphology, number of mucus-producing cells and expression level of coronavirus receptors in three age groups of pigs. Villus height and crypt depth increased with age from 3 days to 3 months in duodenum and ileum but not in mid-jejunum, where the villus height decreased from 580 µm at 3 days to 430 µm at 3 months. Enterocyte length-to-width ratio increased from 3 days to 3 months in all intestinal regions. The number of mucus-producing cells increased with age in the intestinal villi and crypts. The Brunner’s glands of the duodenum contained the highest concentration of mucus-producing cells. The expression of coronavirus receptor APN was highest in the small intestinal villi at all ages. DPP4 expression slightly decreased over time in jejunum and ileum; it was highest in the ileal villi of 3-day-old piglets (70.2% of cells). ACE2 and TMPRSS2 positive cells increased with age in jejunal and ileal crypts and were particularly dominant in the ileal crypts (> 45% of cells). Except for the expression of DPP4 in the jejunum and ileum of young pigs, the expression pattern of the selected coronavirus receptors was very different and not correlated with the age-dependent susceptibility to viral infections. In contrast, the number of mucus-producing cells increased over time and may play an essential role in protecting enteric mucosae against intestinal viruses.

## Introduction

The pig industry faces many challenges from pathogens causing viral gastroenteritis resulting in significant economic losses [1]. Among the viruses, porcine coronaviruses, mainly transmissible gastroenteritis virus (TGEV) and porcine epidemic diarrhea virus (PEDV) cause high morbidity and mortality in pig farms all over the world [2]. Both viruses belong to the genus *Alphacoronavirus* of the family *Coronaviridae*. Additionally, porcine deltacoronavirus (PDCoV) of genus *Deltacoronavirus* is another important diarrhea-causing virus in pigs [3]. Coronaviruses are enveloped, single-stranded positive-sense RNA viruses which undergo significant genetic changes due to frequently occurring mutations in their large genome of 25–30 kDa and recombination [4, 5]. These swift genetic changes are illustrated by the emergence of new coronaviruses [6, 7]. The spike protein of these viruses is mainly involved in host-cell interactions and tissue tropism. The S1 subunit of the spike protein is primarily involved in receptor recognition, while the S2 subunit mediates membrane fusion [8, 9].

PEDV, TGEV and PDCoV infect enterocytes of small intestines in pigs of all ages. PEDV antigens are also found in large intestines except for the rectum [10]. The clinical signs, including diarrhea, vomiting, dehydration and high mortality, are very severe in young piglets during their first days of life. The mortality rate in 1 to 3-day-old piglets without lactogenic immunity can reach 70–100% [11]. The clinical signs become less pronounced with increasing age. Studies on TGEV titer in tissues of different ages of pigs suggest a higher infection rate in 3-day-old pigs than 3-week-old pigs 3–4 days post-infection [12, 13]. Similarly, a study on experimental infection of 2–7 day-old pigs and 2–12 week-old pigs with PEDV found neutralizing antibodies in older pigs. In comparison, younger pigs died after 4 days post-infection [14]. PDCoV infection is also more prevalent and severe in neonatal pigs [15]. To achieve a short breeding period and efficient production, the weaning period is reduced to 21–28 days in commercial pig farms, leading to an early disappearance of the protection by lactogenic immunity. This renders pigs vulnerable to intestinal infections and compromises the young pig’s gut health [16, 17].

Age-related morphological changes in the intestine affect dietary absorption and enteric infections [18]. A recent study on the intestinal barrier function found that young pigs (<1 week of age) have less developed epithelial cells than older pigs based on the production of MUC2, ck18, and lysozymes. Furthermore, the virus-entry facilitating molecules like occludin and porcine APN were also higher in younger pigs [19]. Villus height and crypt depth are other vital hallmarks of intestinal development and health [20]. The morphological data of these factors in different ages of pigs are scarce and may provide a possible link between age-dependent susceptibility and intestinal development.

Mucus is the primary interface between nutrients, pathogens and host defenses. In the small intestine, a single, relatively thinner mucus layer (25.9 ± 11.8 μm in duodenum to 31.0 ± 15.7 μm in ileum) is easily penetrable by nutrients. Conversely, the colon has two distinguished mucus layers: an inner layer adherent to the epithelium and a loose outer layer with thickness reaching up to 35.1 ± 16.0 μm in the descending colon [21, 22]. The mucus pore size also depends on the intestine region increasing in size from the small intestine to the colon. The mucus mainly comprises mucins and other immunomodulatory molecules and differs in different intestinal areas [23]. Mucins have a protein backbone combined with O-linked oligosaccharide modifications [24]. They are broadly classified as membrane-bound and gel-forming mucins. Among these, gel-forming MUC2, MUC5AC, MUC5B and MUC6 mainly undergo homo-oligomerization by forming disulfide bonds at their cysteine-rich N- and C-terminals giving mucus its viscoelastic properties [25, 26]. MUC2 is the principal gel-forming mucin in the intestines that contributes to the formation of the mucus barrier [27]. MUC5AC is typically produced in the stomach but can also be upregulated within the intestines during parasitic enteric infections [28]. MUC5B expression is observed in the colon. MUC6 is expressed in the stomach and duodenum [29, 30]. MUC7, a low molecular weight mucin found in saliva, is often categorized separately as it does not play a role in providing viscoelastic properties [31]. The mucus layer protects the intestinal mucosa against chemical damage from intraluminal host proteases and the invasion of pathogens [32].

Coronaviruses enter enterocytes using transmembrane receptors. Aminopeptidase N (APN, CD13) is a type II transmembrane protein containing 967 amino acids with a large ectodomain. It is expressed in mammalian tissues, mainly the intestines and nervous system and cleaves amino acids from the amino terminus of peptides [33, 34]. It is involved in many important physiological processes such as removal of N-terminal amino acids from protein substrates during digestion in pigs [35], blood pressure regulation and pain sensation, tumor angiogenesis and metastasis, and immune cell chemotaxis [36]. It is widely studied for its role as a functional receptor for coronaviruses like human coronavirus 229E, TGEV, porcine respiratory coronavirus (PRCV), and infectious bronchitis virus (IBV) [37–41]. Recent studies have indicated that APN is not the sole entry receptor for TGEV and PEDV [42–44]. Interaction of PDCoV with APN is also studied in recent years. Since PDCoV is able to infect APN-knockout pigs, the role of APN as a functional receptor is questioned for enteric infections [35]. Additional receptors have also been identified for several other coronaviruses. Dipeptidyl peptidase 4 (DPP4, CD26) is a serine exopeptidase that regulates neuropeptides, chemokines, and peptide hormones by cleaving X-proline dipeptides from the N-terminus [45]. With a short cytoplasmic and transmembrane chain (20–30 amino acids) and large ectodomain (~730 amino acids), it is expressed on endothelial cells and lymphocytes, with a soluble form also found in blood and lymph. It plays a significant role in glucose homeostasis [46]. It was identified as a primary cellular receptor for the highly pathogenic Middle-East respiratory syndrome coronavirus (MERS-CoV) in 2013 [47]. Simulation molecular docking studies have recently investigated its weaker interaction with SARS-CoV2 [48, 49]. Angiotensin-converting Enzyme 2 (ACE2) is a type I membrane protein that converts angiotensin 2 to angiotensin 1–7. It is a 120 kDa metallo-carboxypeptidase expressed in the intestines, kidneys, testes, gall bladder and heart [50]. Its role as the primary receptor for SARS-CoV and SARS-CoV2 has been established [51, 52]. Transmembrane Protease Serine 2 (TMPRSS2) is a type II transmembrane, trypsin-like endopeptidase expressed in the respiratory system and small intestines [53, 54]. Its role in priming the spike protein of some SARS-CoV and SARS-CoV2 and in assisting the virus-cell fusion has been demonstrated [51, 55]. To our knowledge, a detailed expression pattern of coronavirus receptors in porcine intestines is currently unavailable.

This study was designed (i) to do intestinal morphometry at tissue and cellular levels; (ii) to quantitate the number of mucus-producing cells and (iii) to determine the expression pattern of the coronavirus receptors APN, DPP4, ACE2, and TMPRSS2 in the duodenum, jejunum, ileum and colon of 3 day, 3 week and 3 month-old pigs. This may help in better understanding the age-dependent resilience to enteric viral infections.

## Materials and methods

### Pigs

Pigs at 3 days, 3 weeks and 3 months of age (TN70 × Belgian Piëtrain) from a healthy conventional pig farm were used for the present study. At the time of pig collection, no clinical signs were present. Three pigs from each age-group (total *n* = 9) were used to repeat the experiments. The pigs were euthanized with pentobarbital (Kela, Hoogstraten, Belgium) at a dose of 12.5 mg/kg body weight according to standard recommendations of the Ethical Committee of the Faculty of Veterinary Sciences at Ghent University, Belgium.

### Tissue samples

The abdominal cavity was opened and the intestines from the duodenum to the colon were isolated. Three pieces (2 cm long) of the duodenum, mid-jejunum, ileum and colon were collected from each pig. For further histological analysis, one piece from each part was put in a 4% paraformaldehyde solution. For the mucus staining, a second segment was filled with PolyFreeze Tissue Freezing Medium (Sigma) and frozen at −70 °C. For the receptor staining, a third segment was placed in ice-cold Dulbecco’s Modified Eagle Medium (DMEM; Gibco BRL, Merelbeke, Belgium), supplemented with 100 U/mL penicillin, 0.1 mg/mL streptomycin, 0.1 mg/mL gentamycin, 10 µg/mL Fungizone (Sigma) and 10% fetal bovine serum (FBS; Gibco BRL); it was washed thoroughly to get rid of luminal contents and embedded in a methylcellulose medium (ThermoFisher GmbH, Kandel, Germany) and snap-frozen in dry ice with ethanol (−70 °C).

### Histological processing

Tissue samples fixed in 4% paraformaldehyde were sent to the Department of Morphology, Faculty of Veterinary Medicine, Ghent University for histological processing and hematoxylin and eosin staining, as well as Periodic Acid Schiff (PAS) staining [56, 57]. Briefly, the tissues were dehydrated in graded alcohols and embedded in paraffin wax using the STP 120 Microm Tissue Processor and the embedding center EC350-1 and 2 (Microm, Prosan). Cross-sections of 8 µm thickness were cut with the HM 360 Microtome using the Section Transfer System (Microm, Prosan). The sections were stained with hematoxylin/eosin (HE) for general histology using a standard procedure. For goblet cell staining, sections were rehydrated and stained with periodic acid (Servicebio) for 5 min; the slices were then treated with Schiff’s staining (Servicebio) for 30 min under protection from light; after washing with running tap water, the sections were subjected to hematoxylin staining, dehydrated by xylene and mounted.

### Measurement of villus height, crypt depth, and colonic mucosa

Tissues stained with H&E were observed by light microscopy at a 100× magnification (Leica Microsystems GmbH). ImageJ (NIH, US) was used to measure the villus height and crypt depth of small intestines and the crypt depth of the colon for all age groups. The scaling factor of ImageJ was calibrated according to the known micron scale of each image to convert the pixels into a known distance. The villus height was measured from the tip of each villus till the start of the crypt. From each section, the measurements were performed from 5 complete villi or crypts in three randomly selected areas. Similarly, the crypt depth was measured from the end of each villus till the start of the submucosal layer. For the colon, the whole mucosal layer was measured from the luminal surface till the beginning of the submucosal layer.

### Enterocyte morphometry

Tissues stained with H&E were observed by light microscopy at a 630× magnification (Leica Microsystems GmbH). ImageJ (NIH, US) was used to acquire pictures and perform morphometric analysis. The scaling factor of ImageJ was calibrated according to the known micron scale of each image to convert the pixels into a known distance. The length and width of 5 randomly selected enterocytes from 10 neighboring villi and crypts were measured for each intestinal region and age group. The length-to-width ratio was calculated for each reading.

### Quantitation of mucus-producing (PAS^+^) cells

**Figure 1:**
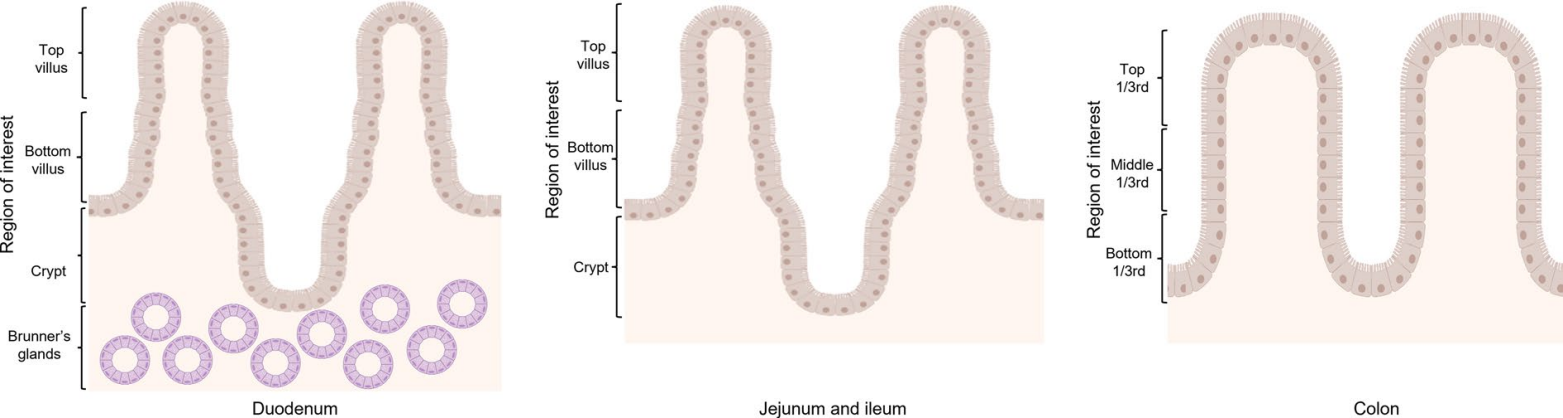
**Regions of interest in duodenum, jejunum, ileum and colon for quantitation of PAS**^**+**^** cells.**

Methodology 1—PAS staining of paraffin sections. The PAS-positive cells (magenta) and the total number of cells (blue nuclei) were counted in 10 neighboring complete villi and crypts from 3 randomly selected regions of each slide/age group at a 200× magnification by light microscopy. Each villus/crypt was divided into three parts for quantification, as shown in Figure 1. In the duodenum, Brunner’s glands were also considered for quantitation. This was repeated for each region of the intestine and age group. For each part, the percentage of positive cells (positive cells/total cells × 100) was recorded for further analysis.

Methodology 2—PAS staining on cryosections fixed on a Blotting-Nylon 66 membrane. Cryosections of 8 µm thickness from Polyfreeze-embedded tissues were made using a CM 1950 cryostat at −20 °C (Leica Biosystems) with a trimming interval of ~100 µm between each section. Sections were loaded onto 3 cm × 1.5 cm pieces of Blotting-Nylon 66 membranes (type B, positive; Sigma). After air drying, Blotting-Nylon 66 membrane pieces were placed on glass slides for stability and fixed by putting 200 μL of 4% paraformaldehyde on each membrane piece for 10 min at room temperature. After two times washing with 200 μL of distilled water for 5 min each, the pieces were allowed to dry. Afterward, 200 μL of 1% periodic acid at room temperature was added for 10 min. Next, the cryosections were dipped 15 × in distilled water. Next, 200 μL of Schiff’s Reagent was added to the cryosections for 20 min at room temperature. Schiff’s Reagent was removed by tilting the cryosections and 200 μL of SO_2_ water (100 mg/mL Na_2_S_2_O_5_ (sodium metabisulfite) in a solution of 1 M HCl) was added for 2 min. The sections were then dipped 20× in distilled water. After drying, the pieces were mounted on glass slides using adhesive tape and labeled accordingly. The total number of PAS^+^ cells was counted on 10 neighboring villi and crypts (Figure 1) using an Olympus SZX7 Stereomicroscope at a 56× magnification. Brunner’s glands were quantitated in the area underneath 10 neighboring villi and crypts in the duodenum. To get values per villus or crypt, the quantitated values were divided by 10. Three random fields per age group were analyzed and average values per villus, crypt or Brunner’s glands were taken. The thickness of the area that contains Brunner’s glands was also measured using ImageJ in the duodenum of all three ages.

### Fluorescence staining

Cryosections of 8 µm thickness were made from the Methocel-embedded frozen tissue samples with a trimming interval of 100 µm between each section. Sections were made using a CM1950 cryostat at −20 °C (Leica Biosystems) and loaded onto 3-aminopropyltriethoxysilane-coated (Sigma-Aldrich, St. Louis, MO, USA) glass slides. The cryosections were fixed in methanol or 4% paraformaldehyde for 20 min at −20 °C or 10 min at 4 °C, respectively. For APN staining, 4% paraformaldehyde-fixed tissue sections were washed once in PBS for 5 min. Subsequently, they were stained with mouse monoclonal (IgG1) anti-porcine APN antibody (1:100, IMM013, Laboratory of Immunology, Ghent University) containing 10% negative goat serum for one h at 37 °C, followed by goat anti-mouse-IgG FITC labeled antibody for one hour at 37 °C. For DPP4, ACE2, and TMPRSS2, methanol-embedded tissue slides were washed once in PBS for 5 min. Subsequently, they were stained with rabbit polyclonal (IgG) anti-human DPP4 antibody (1:50, LS‑A9026-50, LSBio, WA, USA), goat polyclonal (IgG) anti-ACE2 antibody (1:30, AF933, Thermo Fisher Scientific, MA, USA), and goat polyclonal (IgG) anti-TMPRSS2 antibody (1:30, NBP1-20984, Novus Biologicals, CO, USA) containing 10% negative goat or donkey serum for one hour at 37 °C. This was followed by goat anti-rabbit FITC IgG antibody for DPP4 and donkey anti-goat FITC IgG antibody for ACE2 and TMPRSS2 for one h at 37 °C. The nuclei of all slides were stained with Hoechst (10 μg/mL, Invitrogen) for 10 min at 37 °C, washed three times with PBS and mounted with glycerol-DABCO.

### Receptor quantitation

**Figure 2:**
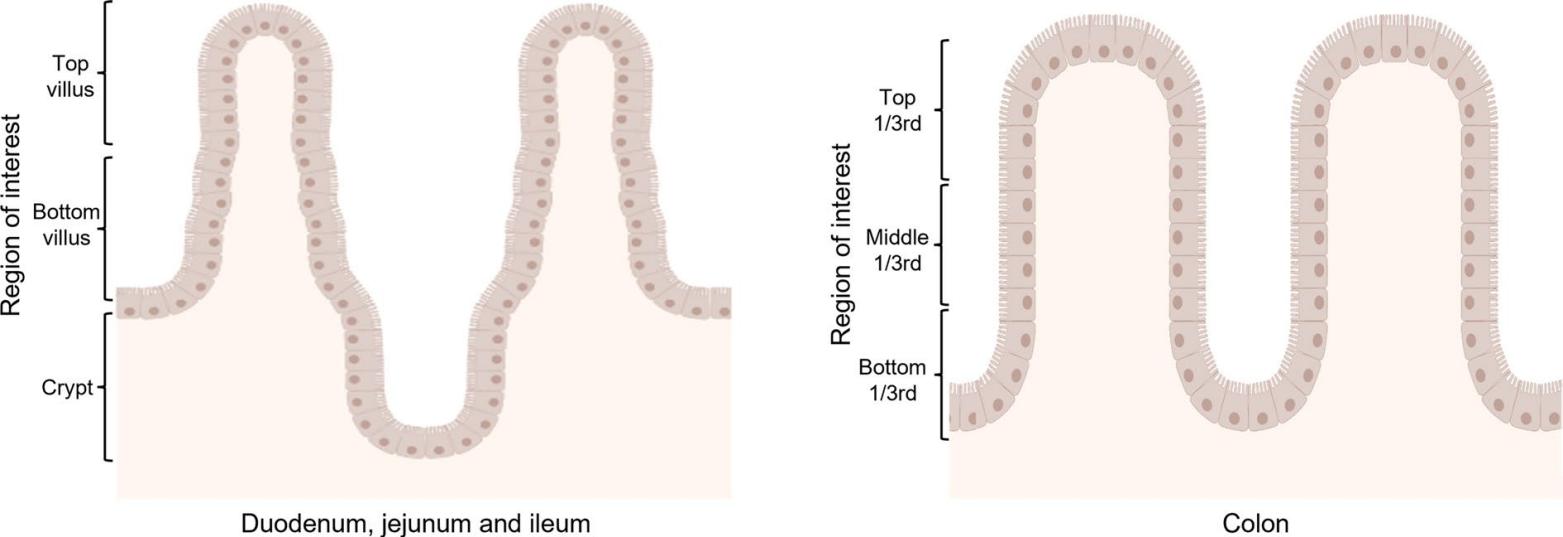
**Regions of interest in duodenum, jejunum, ileum and colon for quantitation of receptor-positive cells**.

From each region of interest of a villus or crypt (Figure 2), the FITC positive cells (green) and the total number of cells (blue nuclei) were counted in 10 consecutive complete villi (small intestine) or crypts (colon) from 3 randomly selected regions of each slide at 200× magnification with a Leica DM RBE fluorescence microscope. This was repeated for each part of the intestine, pig age, and receptor. The average percentage of receptor-positive cells from each location was recorded in MS Excel sheets according to the age of the pig and intestinal region for each receptor.

### Statistical analysis

The analysis of variance (ANOVA) (ordinary one-way/two-way with Tukey’s multiple pairwise comparison) was calculated using GraphPad Prism 9.0 (GraphPad Software, Inc., San Diego, CA, USA). Individual mean values with standard deviation (SD) of three independent experiments were represented as dot plots, and results with *p* values of <0.05 were considered significant.

## Results

### Morphological changes of villi and crypts in small and large intestines with age

**Figure 3:**
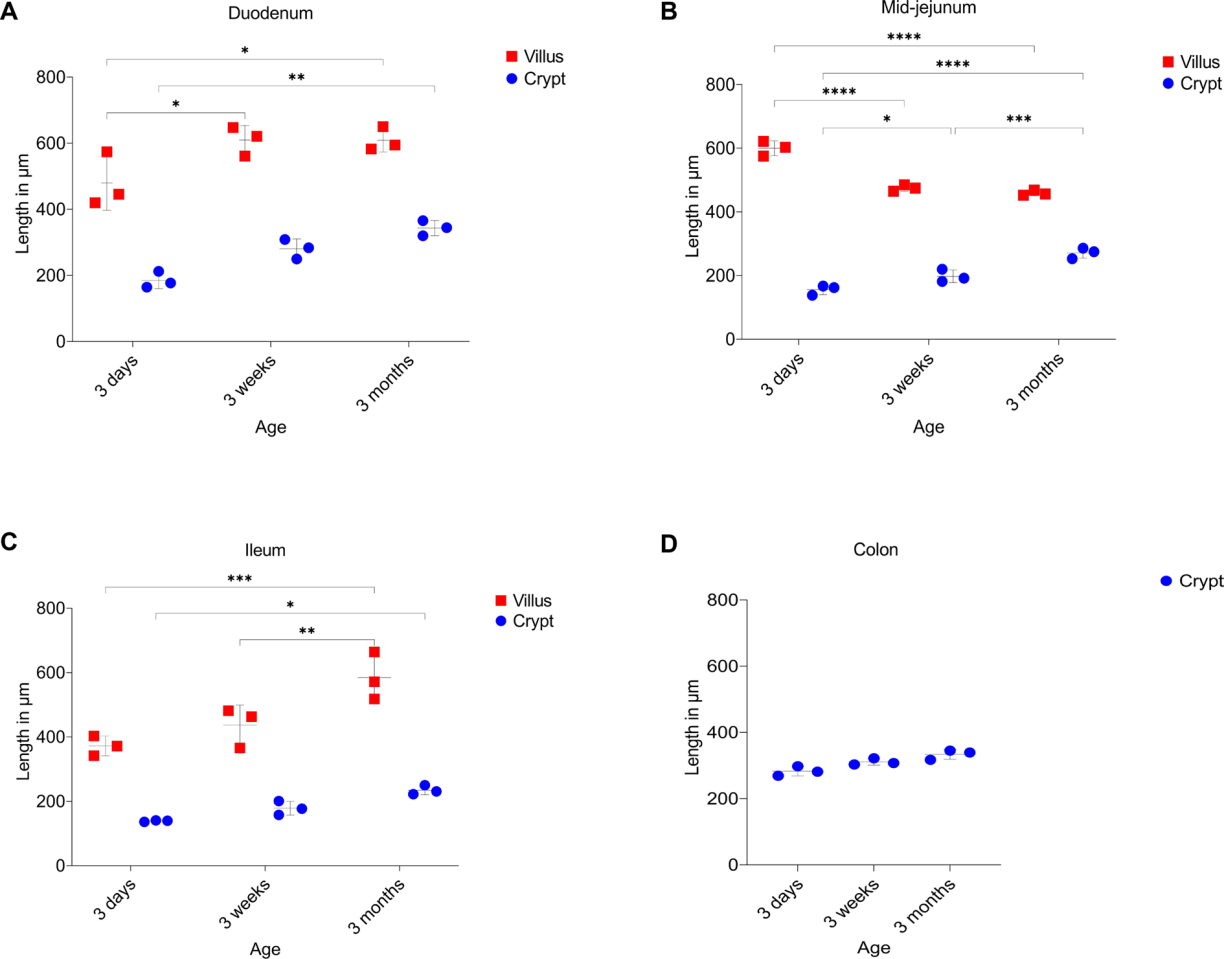
**Measurements for villus height and crypt depth in small intestines [duodenum (A), mid-jejunum (B) and ileum (C)] and crypt depth in colon (D).** Individual mean values ± SD of three animals per age group are plotted. Significant differences between the different age groups (two-way ANOVA with pairwise comparisons) are presented as *p < 0.05, **p < 0.01, ***p < 0.001 or ****p < 0.0001.

The villus height and crypt depth of small intestines (duodenum, mid-jejunum and ileum) and the depth of the colonic crypts were measured for each age group. The mean villus height increased from 3 days to 3 weeks in the duodenum and ileum, whereas it decreased in the mid-jejunum over time (Figures 3A–C). There was a significant difference in mean crypt depths between 3-day-old pigs and 3-month-old pigs in the duodenum (Figure 3A, 184.46 ± 24.82 µm as compared to 344.48 ± 22.89 µm, *p* < 0.01), mid-jejunum (Figure 3B, 149.23 ± 15.40 µm as compared to 270.56 ± 16.79 µm, *p* < 0.0001) and ileum (Figure 3C, 138.87 ± 2.42 µm as compared to 230.81 ± 14.14 µm, *p* < 0.05). The crypt depth in the colon slightly increased with age, but the differences were not significant (Figure 3D, *p* > 0.05). In general, villus height increased with age in the intestines except for the mid-jejunum. Crypt depths also increased with age in all intestinal areas.

### Changes in the morphology of epithelial cells with age

**Figure 4:**
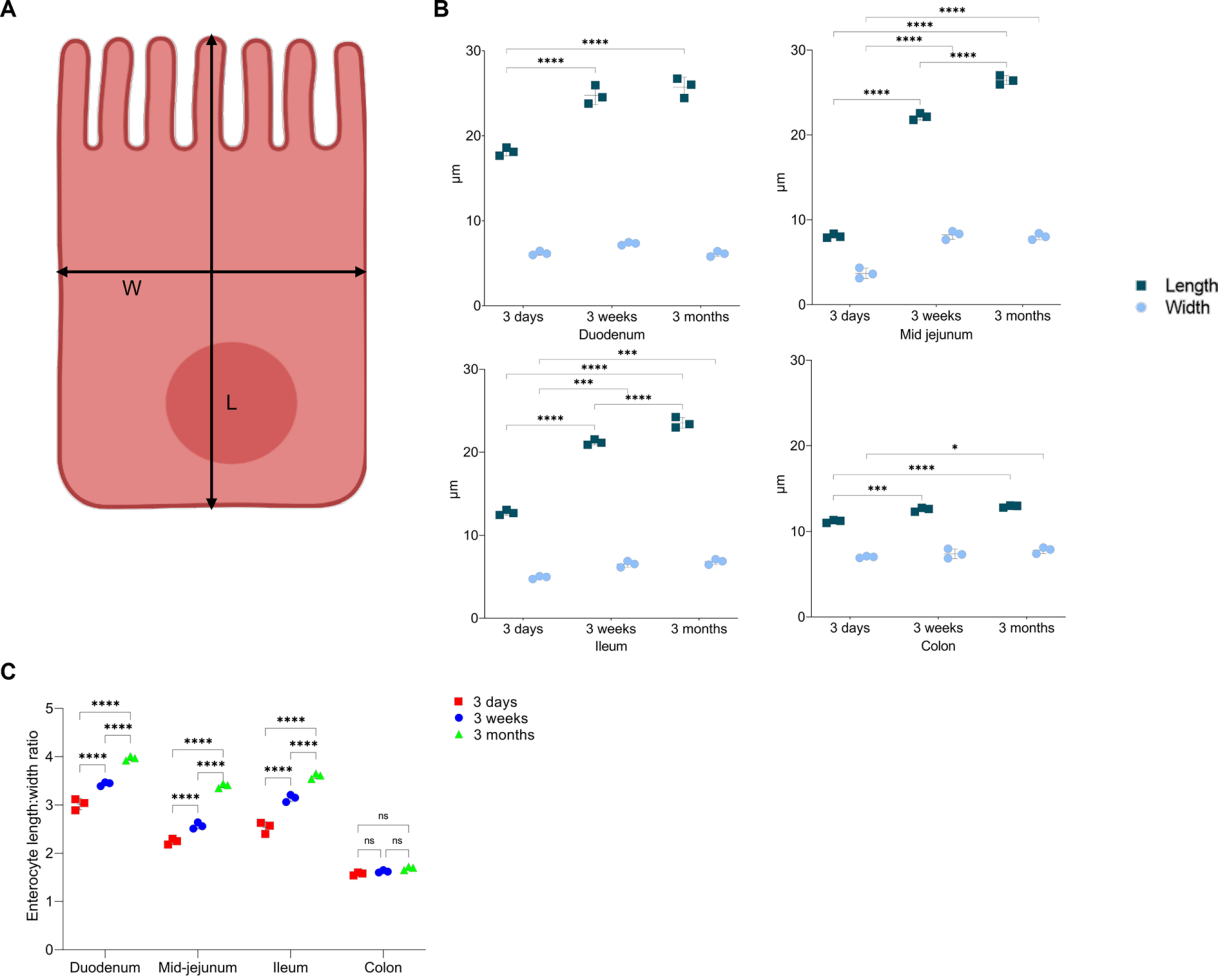
**Porcine enterocyte length to width ratio in different intestinal regions and ages.** **A** Schematic diagram of an enterocyte; black lines show the measurements done with ImageJ (**L** = length, **W** = width). **B** Length and width of enterocytes from villi/crypts of each intestinal region and age of pigs were measured with ImageJ. Mean values were plotted according to the intestinal region and age of the pig. **C** Enterocyte length to width ratio was plotted for each intestinal region and age. Individual mean values ± SD of three animals per age group are plotted. Significant differences between age groups (two-way ANOVA with pairwise comparisons) are presented as *p < 0.05, ***p < 0.001, ****p < 0.0001, or ns (not significant).

The length and width of enterocytes from each intestinal segment and age group were measured by ImageJ, as shown in the schematic diagram (Figure 4A). The mean enterocyte length in the duodenum of 3-week and 3-month-old pigs was significantly longer than that of 3-day-old pigs (Figure 4B; 24.8 ± 1.09 µm and 25.7 ± 1.16 µm versus 18.1 ± 0.47 µm, *p* < 0.0001). In mid-jejunum, the mean enterocyte length increased significantly from 3 days to 3 weeks to 3 months (Figure 4B; 8.1 ± 0.24 µm versus 22.1 ± 0.39 µm versus 26.5 ± 0.53 µm, *p* < 0.0001). A similar trend was seen in the mean enterocyte length of ileum (Figure 4B; 12.7 ± 0.31 µm versus 21.2 ± 0.33 µm versus 23.5 ± 0.64 µm, *p* < 0.0001). For the mean enterocyte width, there was a significant increase from 3 days to 3 weeks in mid-jejunum (Figure 4B; 3.7 ± 0.61 µm versus 8.2 ± 0.51 µm, *p* < 0.0001) and ileum (Figure 4B; 4.9 ± 0.18 µm versus 6.5 ± 0.36 µm, *p* < 0.001). In the colon, mean enterocyte lengths became significantly longer from 3 days to 3 weeks and 3 months (Figure 4B; 11.2 ± 0.17 µm versus 12.6 ± 0.23 µm versus 12.9 ± 0.12 µm, *p* < 0.001). The length-to-width ratio was plotted according to the intestinal region and age (Figure 4C). The mean values of ratio increased in all parts of the small intestines with age. In the duodenum, the length–width ratio increased from 3 ± 0.18:1 in 3-day-old pigs to about 4 ± 0.05:1 in 3-month-old pigs (*p* < 0.0001). In mid-jejunum, the enterocyte length-to-width ratio for 3-month-old pigs (3.3 ± 0.05:1) increased significantly when compared to 3-day-old pigs (2.2 ± 0.06:1) and 3-week-old pigs (2.5 ± 0.07:1). Ileal enterocytes also followed the same trend. Although the colonocytes also increased in length compared to width with age, the difference was not significant (*p* > 0.05). In general, it was demonstrated that the enterocytes in the small intestines of pigs elongated with age.

### Changes in distribution of Goblet cells in small and large intestines with age

**Figure 5:**
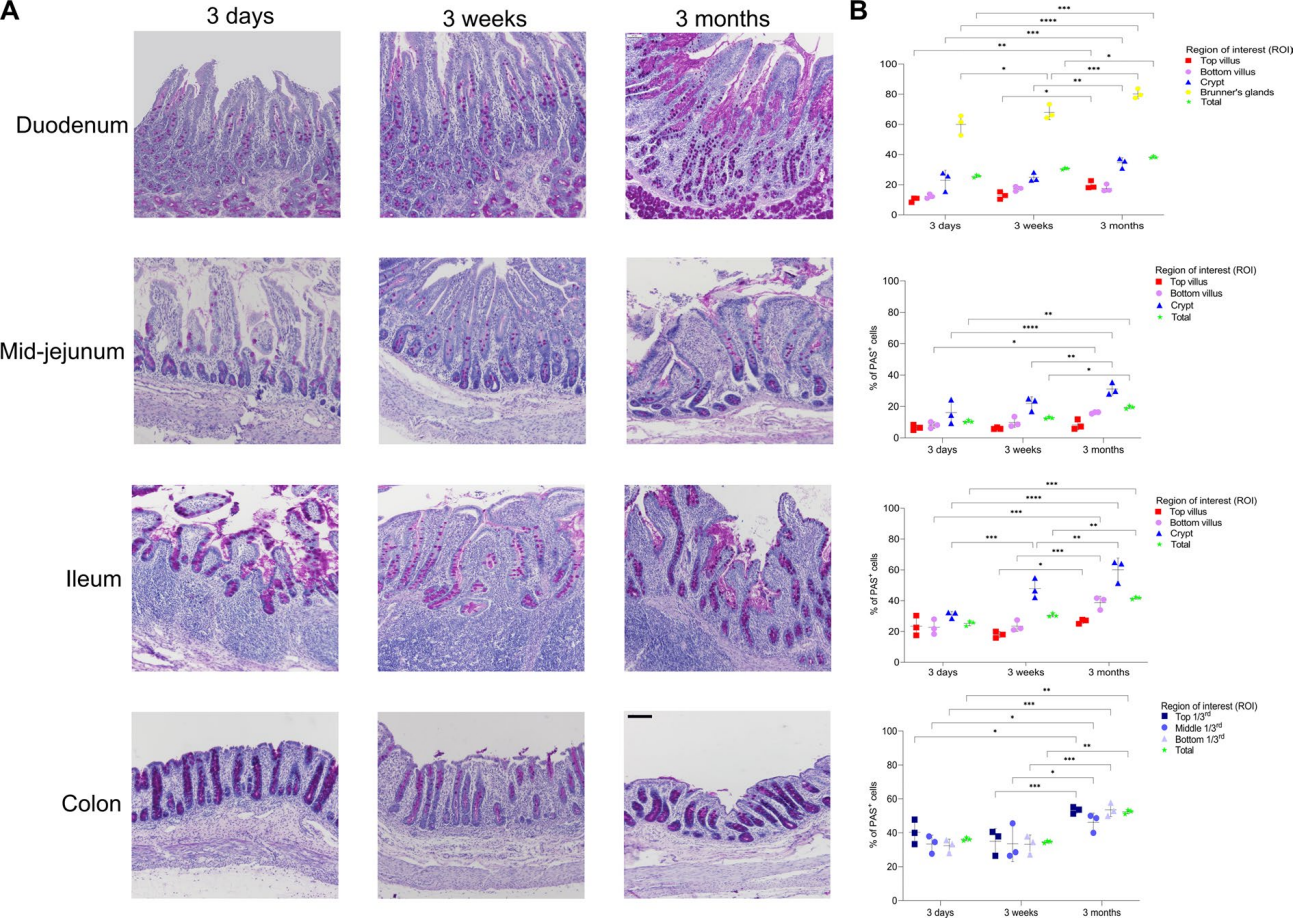
**Quantitation of mucus-producing cells in duodenum, mid-jejunum, ileum and colon of pigs at 3 days, 3 weeks and 3 months of age (paraffin sections; PAS).** **A** Representative pictures of the PAS-stained paraffin tissue sections of the different intestinal regions in the three age groups. **B** Percentage of PAS^+^ cells in duodenum, mid-jejunum, ileum and colon in three age groups. Individual mean values ± SD of three animals per age group are plotted. Significant differences between age groups (two-way ANOVA with pairwise comparisons) are presented as *p < 0.05, **p < 0.01, ***p < 0.001 or ****p < 0.0001. Scale bar: 100 µm.

Methodology 1—PAS staining on paraffin sections. Paraffin-embedded sections of different intestinal segments were stained with PAS to detect Goblet cells (Figure 5A). The mean percentage of PAS^+^ cells (magenta) was determined at different regions of interest (Figure 1), four intestinal parts (duodenum, mid-jejunum, ileum and colon) and three age groups. From the duodenum to the colon, the mean percentage of PAS^+^ cells increased with age in all regions of interest. The highest mean percentage of PAS^+^ cells was observed in the Brunner’s glands of the duodenum and increased significantly with age from 3-day to 3-week to 3 month pigs (Figure 5B; 59.98 ± 6.60% versus 67.92 ± 4.47% versus 80.22 ± 3.16%, *p* < 0.05). Between pigs of 3 days and the pigs of 3 months, the mean percentage of PAS^+^ cells significantly increased in duodenal crypts (Figure 5B; 22.98 ± 6.16% versus 34.59 ± 3.37%, *p* < 0.01), in jejunal crypts (Figure 5B; 16.11 ± 7.67% versus 31.12 ± 3.48%, *p* < 0.001) and ileal crypts (Figure 5B; 31.02 ± 2.12% versus 60.11 ± 7.57%, *p* < 0.001). More PAS^+^ cells were found for all ages in the top 1/3^rd^ of the colonic crypts compared to the middle 1/3^rd^. The mean percentage of PAS^+^ cells in small intestines and colon also increased significantly in 3-month-old pigs compared to younger animals (*p* < 0.05). In general, one can state that the percentage of PAS^+^ cells increased with age along the whole length of the porcine intestines.

**Figure 6:**
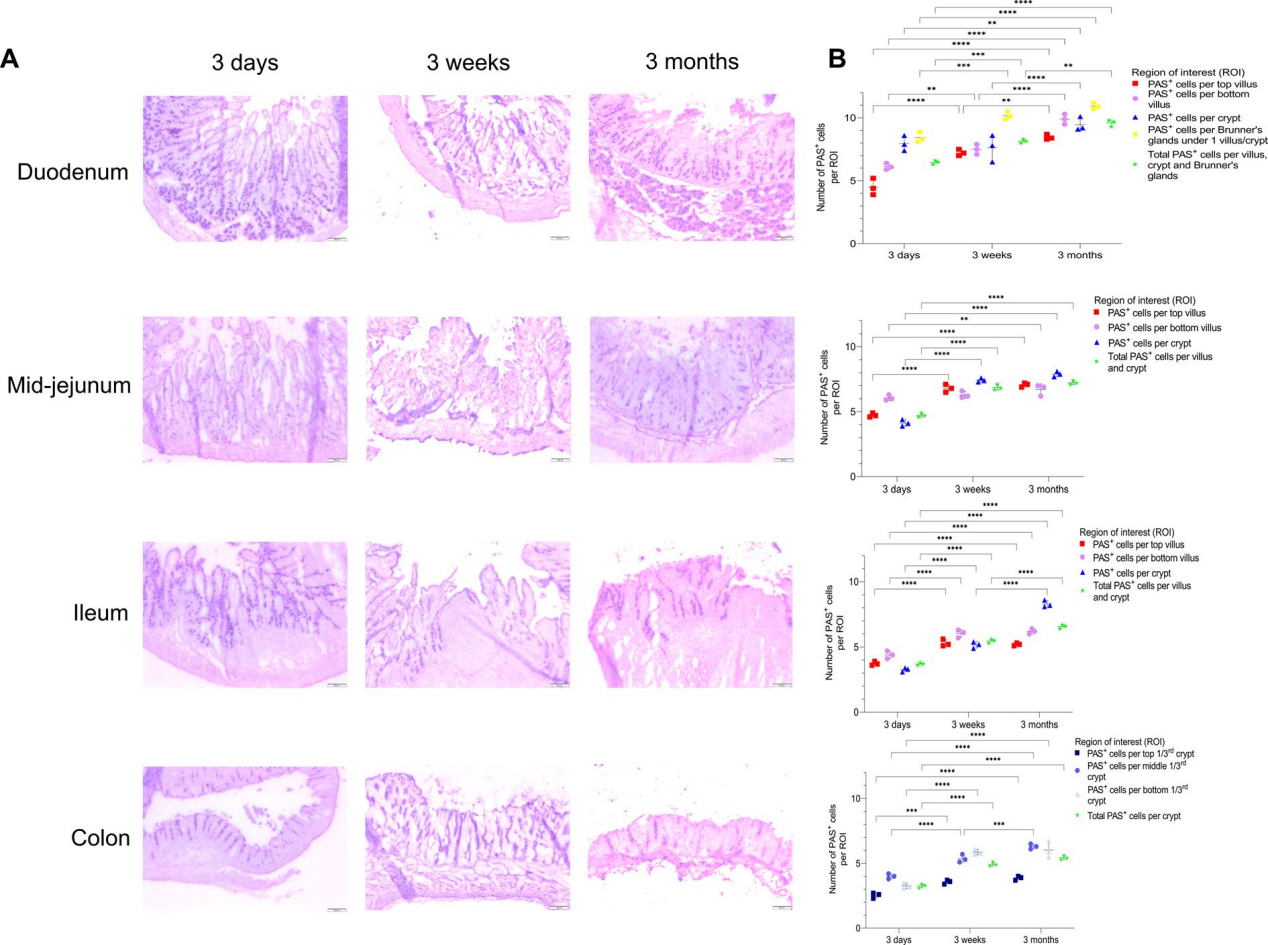
**Quantitation of mucus-producing cells in duodenum, mid-jejunum, ileum and colon of pigs at 3 days, 3 weeks and 3 months of age [cryosections fixed on Blotting-Nylon 66 membranes (type B, positive); PAS].** **A** Representative pictures of the PAS-stained cryosections fixed on Blotting-Nylon 66 membranes (type B, positive) for the different intestinal regions of each age-group. **B** Number of PAS^+^ cells per villus, crypt and Brunner’s glands under one villus and crypt in all parts of interest, in the different intestinal areas of three age groups. Individual mean values ± SD of three animals per age group are plotted. Significant differences in between age groups (two-way ANOVA with pairwise comparisons) are presented as *p < 0.05, **p < 0.01, ***p < 0.001 or ****p < 0.0001. Scale bar: 200 µm.

**Figure 7:**
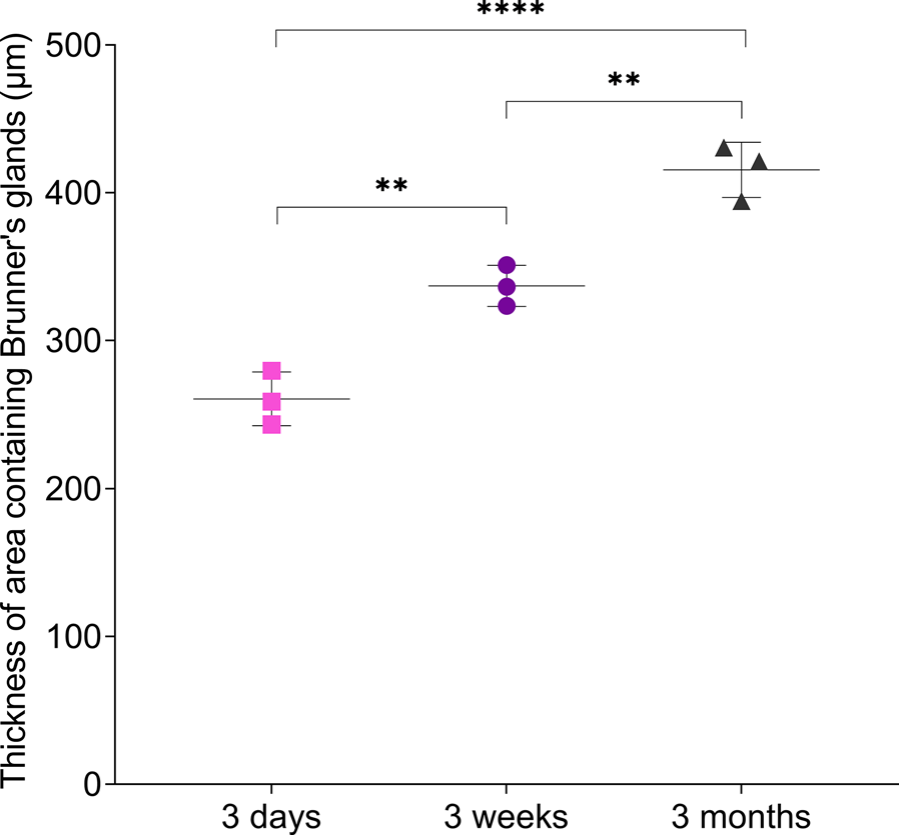
**Thickness of the area containing Brunner’s glands (µm) in the duodenum of pigs at 3 days, 3 weeks and 3 months of age.** Individual mean values ± SD of three animals per age group are plotted. Significant differences in between age groups (ordinary one-way ANOVA with pairwise comparisons) are presented as **p < 0.01 or ****p < 0.0001.

Methodology 2—PAS staining on cryosections fixed to Blotting-Nylon 66 membrane. The mean number of PAS^+^ cells per villus, crypt and Brunner’s gland increased with age. Figure 6A shows representative pictures of the stained cryosections fixed on pieces of Blotting-Nylon 66 membranes (type B, positive). Figure 6B shows the average number of PAS^+^ cells (magenta) per villus, crypt and Brunner's glands present under one villus and crypt for each animal and age-group. The number of PAS^+^ cells per villus, crypt and Brunner’s glands under one villus and crypt increased with age in all intestinal regions (Figure 6B). The mean number of PAS^+^ cells in the Brunner’s glands of the duodenum under each villus and crypt was the highest and increased significantly with age (Figure 6B; 8.43 ± 0.42 versus 10.18 ± 0.31 versus 10.93 ± 0.25, *p* < 0.001). The mean values for thickness of the area containing Brunner’s glands also increased significantly from 3 days to 3 weeks to 3 months (Figure 7; 260.61 ± 18.18 µm versus 337.06 ± 13.90 µm versus 415.57 ± 18.73 µm, *p* < 0.01). At the top of the villus of duodenum, jejunum and ileum, there was a significant increase of mean number of PAS^+^ cells from 3 days to 3 months (Figure 6B; 4.50 ± 0.66 versus 8.48 ± 0.21 in duodenum, 4.73 ± 0.15 versus 7.08 ± 0.15 in mid-jejunum and 3.73 ± 0.15 versus 5.20 ± 0.10 in ileum, *p* < 0.0001). At the bottom of the villus of the duodenum, the mean number of PAS^+^ cells increased with age (Figure 6B; 6.13 ± 0.25 versus 7.50 ± 0.40 versus 9.90 ± 0.40, *p* < 0.01). In the ileal crypt, there was also a significant increase of the mean number of PAS^+^ cells from 3 weeks to 3 months (Figure 6B; 5.18 ± 0.25 versus 8.30 ± 0.27, *p* < 0.001). All regions of interest in the colon of 3-day-old pigs showed significantly lower numbers of PAS^+^ cells per colonic crypt compared to older ages (*p* < 0.01). In general, the number of PAS^+^ cells per villus, crypt and Brunner’s glands increased with age along the whole length of the porcine intestines. The trend was similar in both cryosections fixed on Blotting-Nylon 66 membranes (type B, positive) and paraffin sections.

### APN expression in small and large intestines

**Figure 8:**
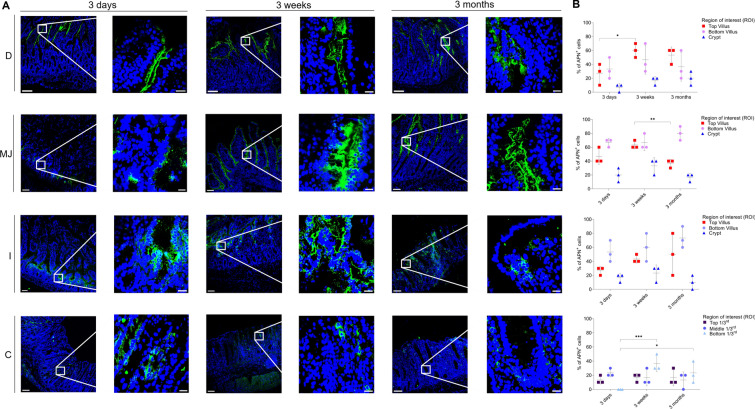
**Expression pattern of APN in small and large intestines.** D = duodenum, MJ = mid-jejunum, I = ileum, C = colon. **A** The expression of APN (green, FITC) was expressed as number of positive epithelial cells on the total number of epithelial cells (%). **B** The percentage of APN^+^ cells in each villus (top and bottom) and crypt (small intestine) and three parts of each crypt (top 1/3^rd^, middle 1/3^rd^ and bottom 1/3^rd^) (colon) was measured. Individual mean values ± SD of three pigs per age group were plotted according to the intestinal region and age. Significant differences between the different age groups (two-way ANOVA with pairwise comparisons) are presented as *p < 0.05, **p < 0.01 or ***p ≤ 0.001. Scale bar: 125 µm and 25 µm.

The expression pattern of APN in different intestinal regions of all three age groups was analyzed by fluorescence microscopy (Figure 8A). The highest expression was observed at the bottom of the villi of each intestinal region and age, followed by the top of the villi (Figure 8B). The expression pattern was variable between age groups, with a significant increase in the mean values of top duodenal villus of the 3-week-old pigs (60.32 ± 10.63%), compared to that of the 3-day-old pigs (26.67 ± 15.26%) (*p* < 0.05). In the jejunal top of the villus, the mean expression increased from 3 days (46.67 ± 11.55%) to 3 weeks (63.34 ± 5.75%) but significantly decreased again in 3-month-old pigs (36.68 ± 5.75%) (*p* < 0.01). APN mean expression increased with age in ileal villi for all ages but dropped in the ileal crypts of 3-month-old pigs (10.02 ± 9.60%) compared to 3-week-old pigs (23.34 ± 11.57%). The mean expression in the colon was lower than in the small intestines, with a significant increase in the bottom crypt from 3 days (nil) to 3 weeks (36.68 ± 11.55%) (*p* < 0.001) and a considerable decrease in 3-month-old pigs (23.34 ± 15.26%) (*p* < 0.05). This shows that APN is highly expressed in all intestinal regions and that the expression pattern is relatively constant over age.

### DPP4 expression in small and large intestines

**Figure 9:**
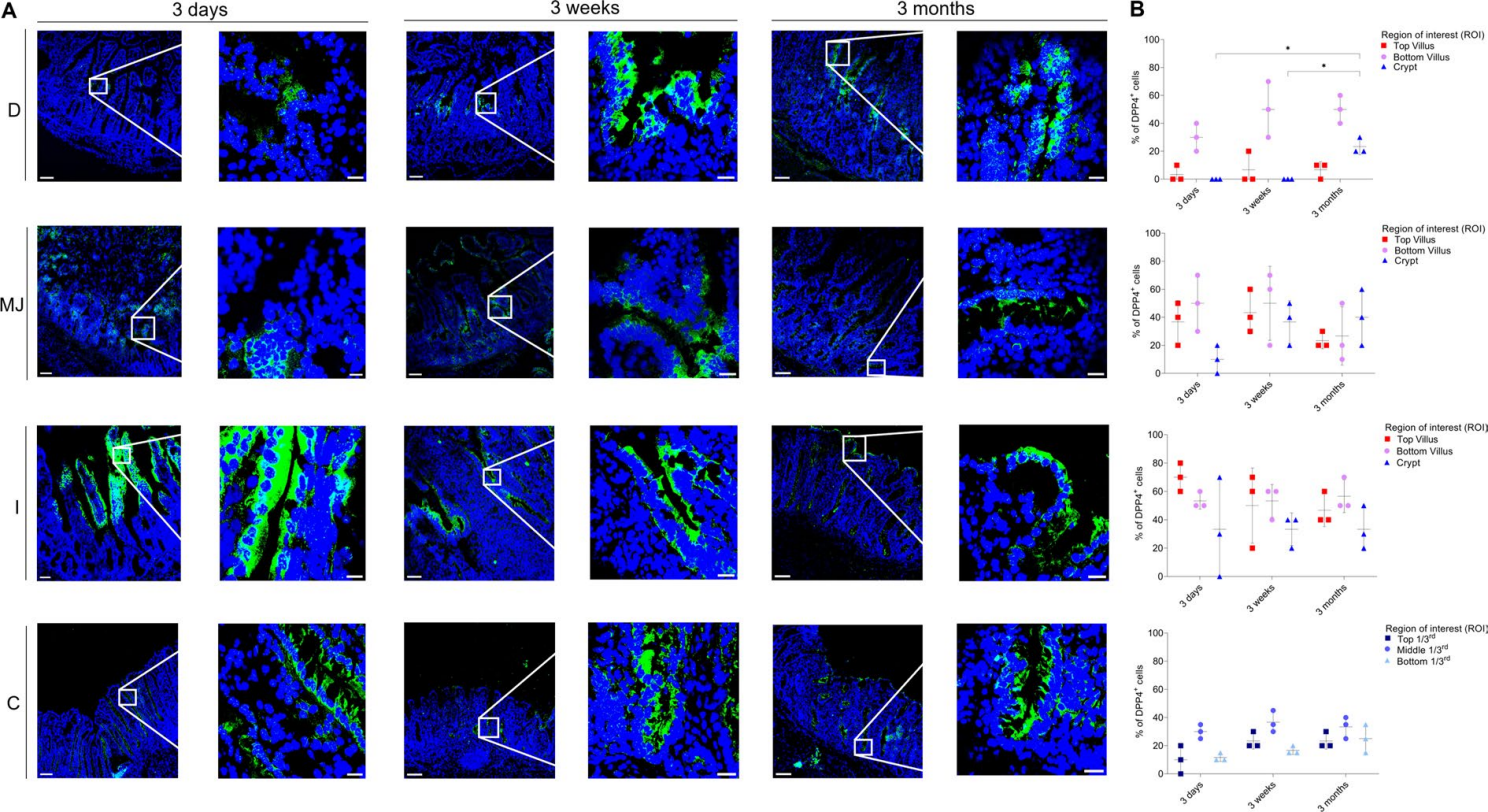
**Expression pattern of DPP4 in small and large intestines.** D = duodenum, MJ = mid-jejunum, I = ileum, C = colon. **A** The expression of DPP4 (green, FITC) was expressed as number of positive epithelial cells on the total number of epithelial cells. **B** The percentage of DPP4^+^ cells in each villus (top and bottom) and crypt (small intestine) and three parts of each crypt (top 1/3^rd^, middle 1/3^rd^ and bottom 1/3^rd^) (colon) was measured. Individual mean values ± SD of three pigs per age group were plotted according to the intestinal region and age. Significant differences between the different age groups (two-way ANOVA with pairwise comparisons) are presented as *p < 0.05, **p < 0.01 or ***p ≤ 0.001. Scale bar: 125 µm and 25 µm.

The expression pattern of DPP4 in different intestinal regions of all three age groups was analyzed by fluorescence microscopy (Figure 9A). The highest expression was seen in the ileum for all ages, followed by the jejunum (Figure 9B). In the duodenal crypts, only 3-month-old pigs expressed DPP4 (22.34 ± 5.77%). In jejunal crypts, 3-day-old pigs showed a much lower expression (10.31 ± 9.76%) in comparison to older ages (>35%). In the ileum of 3-day-old pigs, the top villi showed the highest expression (70.09 ± 10.23%) compared to older ages (<50%). In colonic crypts, all ages expressed DPP4, particularly in the middle 1/3^rd^ portion (>24%). This exhibits that the expression pattern of DPP4 slightly decreases (not significantly, *p* > 0.26) over age.

### ACE2 expression in small and large intestines

**Figure 10:**
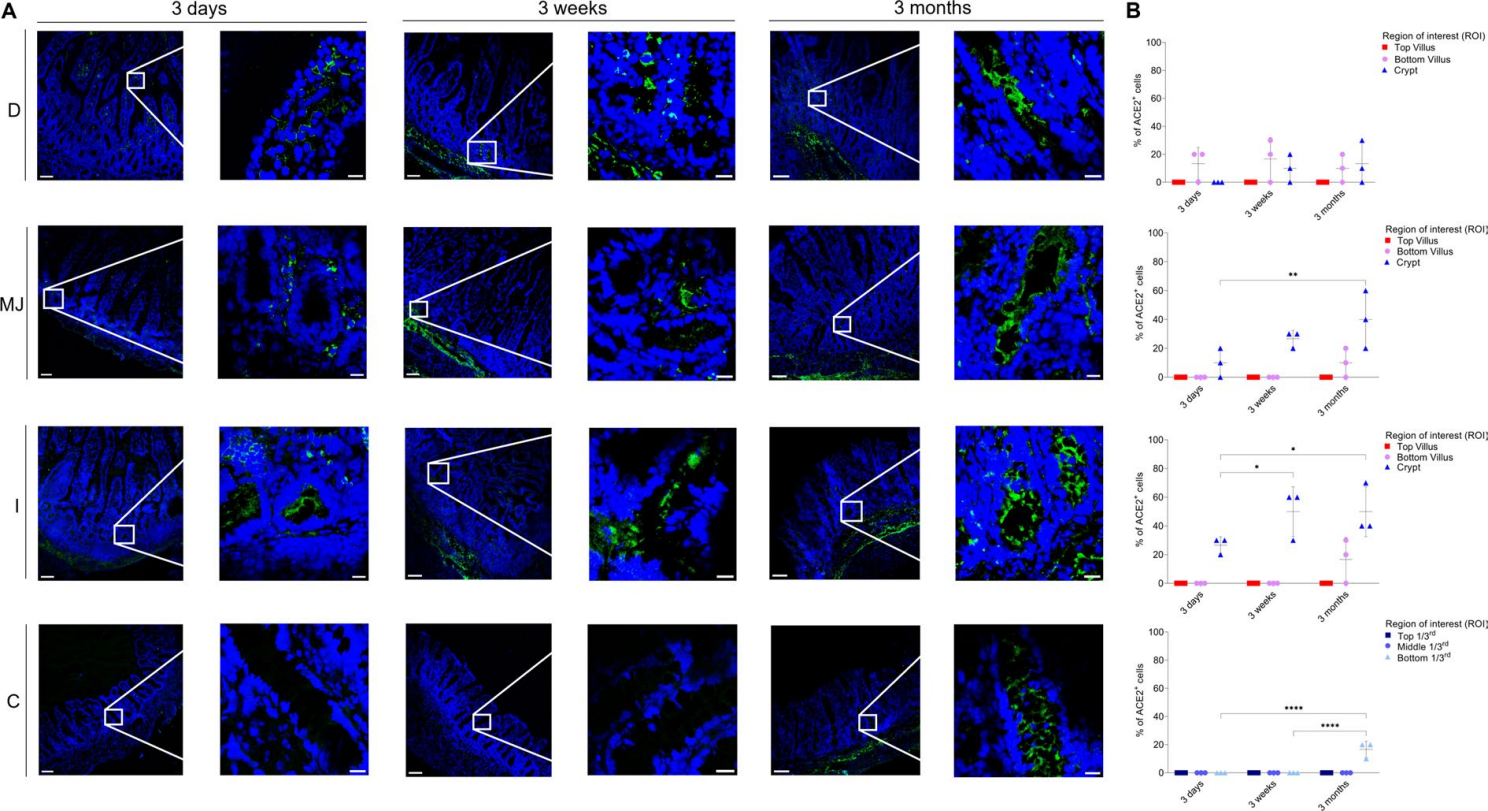
**Expression of ACE2 in small and large intestines.** D = duodenum, MJ = mid-jejunum, I = ileum, C = colon. **A** The expression of ACE2 (green, FITC) was expressed as number of positive epithelial cells on the total number of epithelial cells. **B** The percentage of ACE2^+^cells in each villus (top and bottom) and crypt (small intestine) and three parts of each crypt (top 1/3^rd^, middle 1/3^rd^ and bottom 1/3^rd^) (colon) was measured. Individual mean values ± SD of three pigs per age group were plotted according to intestinal region and age. Significant differences between the different age groups (two-way ANOVA with pairwise comparisons) are presented as *p < 0.05, **p < 0.01 or ***p ≤ 0.001. Scale bar: 125 µm and 25 µm.

The expression pattern of ACE2 in different intestinal regions of all three age groups was analyzed by fluorescence microscopy. The ACE2-positive cells were dominant in small intestinal crypts, particularly in the ileum and were not present on the top of villi (Figure 10A). In the duodenum of 3-day-old pigs, ACE2 was not expressed. A significant difference between mean expression levels in jejunal crypts of 3-month-old pigs (40.35 ± 18.07%) was observed, compared to 3-day-old pigs (10.12 ± 9.56%) (Figure 10B, *p* < 0.01). Similarly, ileal crypts of 3-day-old pigs also showed a significantly lower expression (26.67 ± 5.75%) when compared to older ages (>45%) (*p* < 0.05). In the colon, only 3-month-old pigs expressed ACE2 in the bottom 1/3^rd^ of the crypts (16.67 ± 5.76%). This shows that the expression of ACE2 in the small intestinal crypts increases with age and is only expressed in the colon at 3 months.

### TMPRSS2 expression in small and large intestines

**Figure 11:**
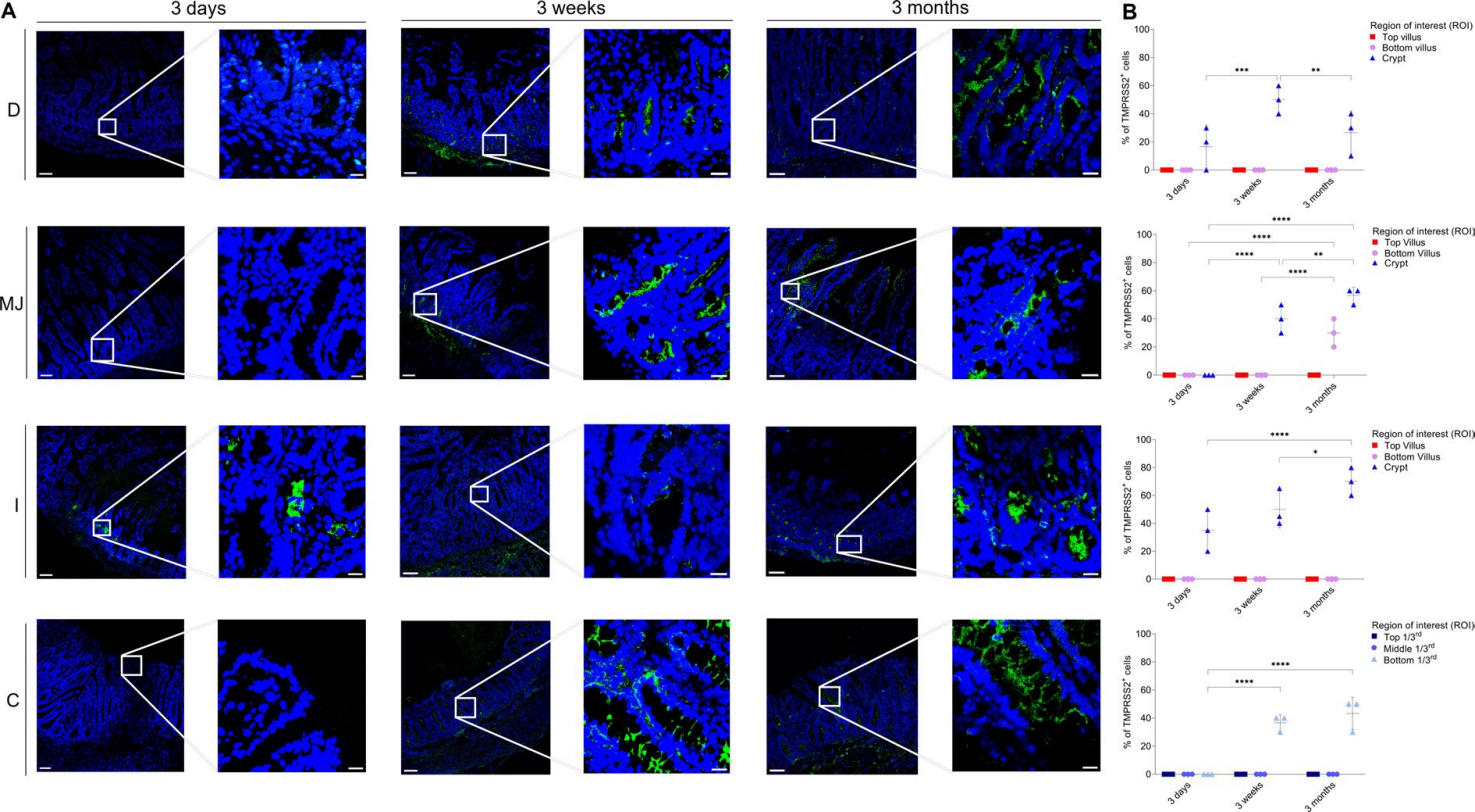
**Expression pattern of TMPRSS2 in small and large intestines.** D = duodenum, MJ = mid-jejunum, I = ileum, C = colon. **A** The expression of TMPRSS2 (green, FITC) was expressed as number of positive epithelial cells on the total number of epithelial cells. **B** The percentage of TMPRSS2^+^ cells in each villus (top and bottom) and crypt (small intestine) and three parts of each crypt (top 1/3^rd^, middle 1/3^rd^ and bottom 1/3^rd^) (colon) was measured. Individual mean values ± SD of three pigs per age group were plotted according to intestinal region and age. Significant differences between the different age groups (two-way ANOVA with pairwise comparisons) are presented as *p < 0.05, **p < 0.01 or ***p ≤ 0.001. Scale bar: 125 µm and 25 µm.

The expression pattern of TMPRSS2 in different intestinal regions of all three age groups was analyzed by fluorescence microscopy. Similar to the expression pattern of ACE2, TMPRSS2-positive cells were also dominant in the small intestinal crypts for all ages (Figure 11A). In duodenal crypts, the mean number of TMPRSS2 positive cells significantly increased from 3 days (16.67 ± 15.26%) to 3 weeks (50.36 ± 9.87%) but decreased again at 3 months (26.67 ± 14.87%) (Figure 11B, *p* < 0.01). The duodenum and colon of 3-day-old pigs did not express TMPRSS2. The expression level of TMPRSS2 increased from 3 weeks (50.01 ± 13.23% and 36.67 ± 5.79%) to 3 months (71.58 ± 11.76% and 43.34 ± 11.55%) in the ileal crypts and the bottom 1/3^rd^ of the colonic crypts, respectively. From these results, it can be concluded that TMPRSS2 colocalizes very well with ACE2 in the porcine intestines as it does in the conjunctiva and respiratory tract of humans [58, 59].

## Discussion

Enteric viral diseases are a significant concern for the pig industry due to early mortality [60]. Porcine epidemic diarrhea virus (PEDV), transmissible gastroenteritis virus (TGEV), and porcine rotaviruses are the most important viruses causing severe diarrhea leading to death in young piglets [1]. The mechanisms behind these severe infections in young piglets are not well-understood. This study attempted to associate this age-dependent viral susceptibility with changes in the intestinal morphology at tissue and cellular levels, number of mucus-producing cells, and the expression of important coronavirus receptors (APN, DPP4, ACE2, TMPRSS2). Although, DPP4, ACE2 and TMPRSS2 are not yet described as porcine coronavirus receptors, they are known receptors for many other coronaviruses in other species as mentioned above. Recent studies have indicated that some porcine coronaviruses like PEDV and PDCoV do not necessarily need APN for infecting cells [35, 43, 76, 77]; they might use other coronavirus receptors. Thus, this study aimed to show the expression of these known coronavirus receptors in the intestines of different ages of pigs which was not shown before.

Villus height, crypt depth and enterocyte length-to-width ratio were first analyzed in different intestinal regions and age groups. Villus height increased in length with age in the duodenum and ileum but not in the jejunum, where the length of the villi decreased. Crypt depths increased with age in all intestinal regions. In addition, enterocytes in the small intestines of pigs became more elongated with age. The number of mucus-producing cells increased with age along the whole length of the porcine intestines in both paraffin sections and cryosections fixed on Blotting-Nylon 66 membranes (type B, positive). Finally, it was observed that the expression patterns of the selected coronavirus receptors APN, ACE2 and TMPRRS2 in porcine intestines were not correlated with the age-dependent loss of susceptibility. The expression pattern of DPP4 formed an exception to this general rule.

Villus height and crypt depth are important morphological features that change with animal age [61]. It was already reported that after weaning, the villus height of enterocytes in the small intestines generally decreases for a certain period and crypt depth increases [62]. In our study, this trend was only observed in the jejunum, where the villus height decreased from ~580 µm in 3-day-old pigs to about ~470 µm in 3-month-old pigs. Our results agreed with a recent study where the villus height in the jejunum also decreased from ~340 µm in 1-week-old pigs to ~270 µm in 4-week-old pigs [19]. The difference in length between these two studies could be attributed to the different genetic backgrounds of the animals. In the present study, TN70 × Belgian Piëtrain pigs were used. In 2019, a research group identified changes in H3K4me3 enrichment and gene expression patterns in pigs infected with PEDV [63]. As breed variations may affect the gene and receptor expression, we suggest that the genetic makeup of the pigs should also be considered when carrying out age-dependent infection studies. The longer villi during the first weeks of life may be linked to the high demand for nutrients in colostrum and milk of neonatal piglets during the suckling period. When the animals switch to solid feed, damage on the tips of the villi may lead to a shortening of the villi. The increase in the number of mucus-producing cells with age, as observed in the present study, may also be linked with this milk-solid feed switch. Indeed, mucus needs to protect the enterocytes against damages caused by the solid feed. In this context, it would be interesting to additionally examine in the future if the mucus composition changes as well with age.

Entry-mediators mediate viral entry at enterocytes’ apical and basolateral surfaces [44]. The apical and basolateral plasma membranes of polarized enterocytes allow for the site-specific distribution of viral receptors and oriented transport of nutrients and immunoglobulins across the intestinal epithelial cell monolayer [64]. This study analyzed this polarity in terms of enterocyte length-to-width ratio in all intestinal regions and ages of pigs. The enterocytes for all intestinal parts increased in length as compared to width. With age, this elongation of enterocytes gives the cells a more vital polarity and may be linked to a better expression of receptors and more performant digestion. A low polarity, as in the case of 3-day piglets, may lead to less efficient digestion and diarrheal osmotic disorders [65]. Longer villi in the jejunum of young suckling animals may form compensation for this problem. However, the length-to-width ratio is not the only criterion for analyzing enterocyte morphology’s role in viral infections. Tight junctions, adherens junctions and desmosomes between neighboring enterocytes form a tight barrier against chemical and pathogen damage [66]. Other factors like occludin, epidermal growth factor (EGF), cell proliferation, cell-repairing cytokine IL-22 and porcine β-defensin may also regulate the enteric virus replication in enterocytes [67–70]. Expressions of pattern recognition receptors (PRRs) and cytokines in the pig gut along the proximal/distal and the crypt/villus axis [71], may also change with age and should be considered. In addition, decreased levels of gamma globulins, T cells (CD4 and CD8), and low antigen-specific responses in pigs under 3–4 weeks of age may also render young pigs more susceptible to enteric viral infections [72]. A correlation of morphological features and the presence of these innate and adaptive immunological factors in different age groups of pigs can provide a better understanding of the age-dependent susceptibility of pigs against enteric viral infections at a cellular level.

Goblet cells are responsible for mucus production and their percentage among intestinal epithelial cells in humans increases from the small intestine to the colon [73]. This study found similar results from the jejunum toward the colon. The duodenum was not following this trend. The percentage of mucus-producing cells in this part of the small intestine was higher than in the jejunum. In addition, Brunner’s glands, which are only present in the duodenum, contained an increasing number of mucus-producing cells (~60% at 3 days, ~70% at 3 weeks and ~80% at 3 months). Based on the present work, it may be hypothesized that mucus may play a barrier role in older animals against enteric viruses due to increased production with age.

As the fixation process during mucus staining strongly impacts the preservation of the mucus in histological sections [74], a novel method of fixing cryosections on the positively-charged Blotting-Nylon 66 membranes was compared to the traditional PAS staining on paraffin sections. The results corresponded to each other. Besides mucus quantity, mucus quality may also be important in its barrier function. In this context, further studies on the structure and composition of mucus in different regions of intestines and at different ages are necessary, together with the penetration capacity of viral particles. Another study from our lab used atomic force microscopy (AFM) to analyze the pore size of porcine respiratory mucus and single-particle tracking (SPT) to examine the mobility of pseudorabies virus (PRV) [75]. These techniques can also be used to study, at different ages, the pore size of porcine intestinal mucus and the penetration of TGEV and PEDV through the mucus to get an accurate idea about the role of mucus in hindering viral infections in older pigs. Furthermore, the biochemical composition of mucus in the intestines of pigs at different ages of pigs can also provide interesting information on this aspect.

**Figure 12:**
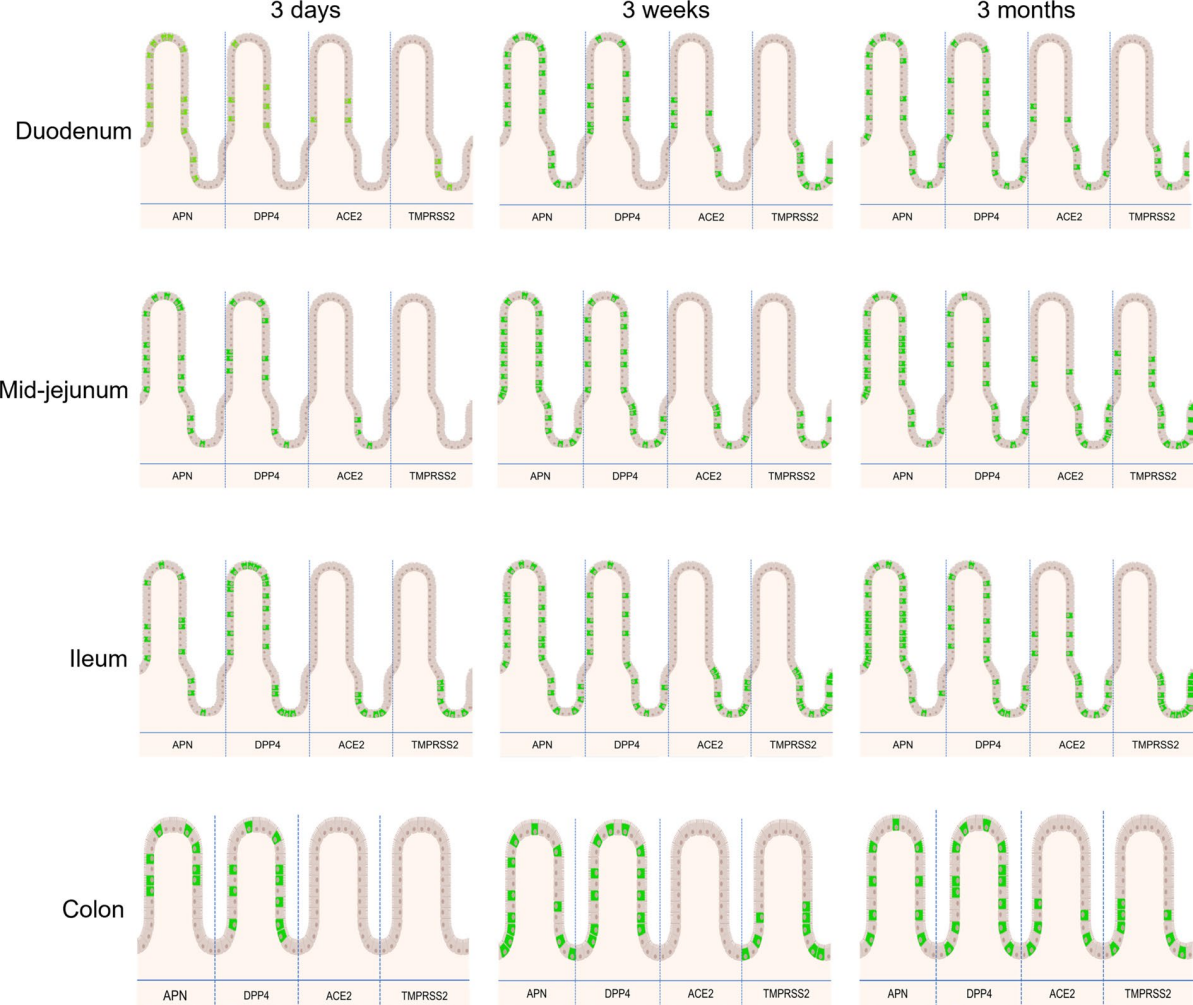
**Schematic overview of expression of coronavirus receptors APN, DPP4, ACE2 and TMPRSS2 in different regions of porcine intestines and ages.** Green represents receptor^+^ cells.

APN is widely expressed in the intestine and is the primary receptor for TGEV [38], as demonstrated by the resistance of APN-knock-out pigs against TGEV [76]. However, many studies, including that of Whitworth et al., suggested that APN is unnecessary for PEDV entry [43, 76, 77]. Similarly, APN is also not a mandatory receptor for PDCoV as the virus is shown to infect APN-knockout pigs [35]. Thus for these viruses, additional unknown receptors are probably present and their identification and role will further elaborate the age-dependent loss of susceptibility in older pigs against PEDV, PDCoV and recently resurfaced Porcine Hemagglutinating Encephalomyelitis Virus (PHEV). The present study hypothesized that a higher expression of APN and other known coronavirus receptors like DPP4, ACE2 and TMPRSS2 in young piglets might play a role in increased enteric infections. The expression pattern of APN, ACE2 and TMPRSS2 in different regions of intestines and the ages of pigs suggested that the lower susceptibility against enteric infections is not correlated to an age-related loss of expression of receptors. The expression pattern in our study was in line with a recent study that studied the distribution of APN, DPP4 and ACE2 in 7 to 10-day-old pigs, which showed a high expression of APN and DPP4 in the small intestine and colon [78]. The expression of receptors was highly variable and, most of the time, increased with age. A schematic overview of the expression of these receptors in porcine intestines of different ages is given in Figure 12.

ACE2 and TMPRSS2 were clearly less expressed in the young pigs. APN was widely expressed in all intestinal regions irrespective of age. DPP4 somewhat supported the hypothesis as it was more expressed in the jejunum and ileum of young ages when compared to older ones, albeit not statistically significant. It is the primary receptor for MERS-CoV [47], and studies have been conducted to see its interaction with SARS-CoV receptor-binding domain, but a relationship could not be found [48, 79]. There are no studies of the involvement of DPP4 with TGEV and PEDV and further exploration is needed.

In conclusion, this study tried to find a cause of the high morbidity and mortality by enteric coronaviruses in young pigs compared to older ones. Shorter villi in the jejunum and increased polarity of enterocytes may be correlated with the reduced severity of intestinal problems with age. Mucus-producing cells also increased with age, suggesting an important role of mucus in protecting older pigs against viral infections. The distribution of coronavirus receptors APN, ACE2 and TMPRSS2 did not go down with age, excluding them as disease-correlated factors; DPP4 formed an exemption to this rule. DPP4, ACE2 and TMPRSS2 are not yet known as porcine coronavirus receptors, but the non-dependency on APN by PEDV and PDCoV make these receptors somewhat relevant and should be explored further with respect to porcine enteric infections.

## References

[1] Zhang Q, Hu R, Tang X, Wu C, He Q, Zhao Z, Chen H, Wu B (2013). Occurrence and investigation of enteric viral infections in pigs with diarrhea in China. Arch Virol.

[2] Saif L, Wang Q, Vlasova A, Jung K, Xiao S (2019). Coronaviruses. Diseases of swine.

[3] Duan C (2022). An updated review of porcine deltacoronavirus in terms of prevalence, pathogenicity, pathogenesis and antiviral strategy. Front Vet Sci.

[4] Vlasova A, Wang Q, Jung K, Langel S, Malik YS, Saif L (2020). Porcine coronaviruses. Emerging and transboundary animal viruses.

[5] Brian D, Baric R (2005). Coronavirus genome structure and replication. Coronavirus replication and reverse genetics.

[6] Boniotti MB, Papetti A, Lavazza A, Alborali G, Sozzi E, Chiapponi C, Faccini S, Bonilauri P, Cordioli P, Marthaler D (2016). Porcine epidemic diarrhea virus and discovery of a recombinant swine enteric coronavirus, Italy. Emerg Infect Dis.

[7] Belsham GJ, Rasmussen TB, Normann P, Vaclavek P, Strandbygaard B, Bøtner A (2016). Characterization of a novel chimeric swine enteric coronavirus from diseased pigs in Central Eastern Europe in 2016. Transbound Emerg Dis.

[8] Hulswit RJG, de Haan CAM, Bosch BJ, Ziebuhr J (2016). Chapter two—coronavirus spike protein and tropism changes. Advances in virus research.

[9] Li F (2015). Receptor recognition mechanisms of coronaviruses: a decade of structural studies. J Virol.

[10] Jung K, Saif LJ (2015). Porcine epidemic diarrhea virus infection: etiology, epidemiology, pathogenesis and immunoprophylaxis. Vet J.

[11] Stevenson GW, Hoang H, Schwartz KJ, Burrough ER, Sun D, Madson D, Cooper VL, Pillatzki A, Gauger P, Schmitt BJ, Koster LG, Killian ML, Yoon KJ (2013). Emergence of porcine epidemic diarrhea virus in the United States: clinical signs, lesions, and viral genomic sequences. J Vet Diagn Invest.

[12] Moon HW, Kemeny LJ, Lambert G, Stark SL, Booth GD (1975). Age-dependent resistance to transmissible gastroenteritis of swine: III. Effects of epithelial cell kinetics on coronavirus production and on atrophy of intestinal villi. Vet Pathol.

[13] Norman JO, Lambert G, Moon HW, Stark SL (1973). Age dependent resistance to transmissible gastroenteritis of swine (TGE). II. Coronavirus titer in tissues of pigs after exposure. Can J Comp Med.

[14] Shibata I, Tsuda T, Mori M, Ono M, Sueyoshi M, Uruno K (2000). Isolation of porcine epidemic diarrhea virus in porcine cell cultures and experimental infection of pigs of different ages. Vet Microbiol.

[15] Zhang J (2016). Porcine deltacoronavirus: overview of infection dynamics, diagnostic methods, prevalence and genetic evolution. Virus Res.

[16] Wang L, Yan S, Li J, Li Y, Ding X, Yin J, Xiong X, Yin Y, Yang H (2018). Rapid communication: the relationship of enterocyte proliferation with intestinal morphology and nutrient digestibility in weaning piglets. J Anim Sci.

[17] Zhong J-F, Wu W-G, Zhang X-Q, Tu W, Liu Z-X, Fang R-J (2016). Effects of dietary addition of heat-killed *Mycobacterium phlei* on growth performance, immune status and anti-oxidative capacity in early weaned piglets. Arch Anim Nutr.

[18] Turner JR (2009). Intestinal mucosal barrier function in health and disease. Nat Rev Immunol.

[19] Yang S, Yang N, Huang X, Li Y, Liu G, Jansen CA, Savelkoul HFJ, Liu G (2022). Pigs’ intestinal barrier function is more refined with aging. Dev Comp Immunol.

[20] Jayaraman S, Thangavel G, Kurian H, Mani R, Mukkalil R, Chirakkal H (2013). *Bacillus subtilis* PB6 improves intestinal health of broiler chickens challenged with *Clostridium perfringens*-induced necrotic enteritis. Poult Sci.

[21] Ermund A, Schütte A, Johansson MEV, Gustafsson JK, Hansson GC (2013). Studies of mucus in mouse stomach, small intestine, and colon. I. Gastrointestinal mucus layers have different properties depending on location as well as over the Peyer’s patches. Am J Physiol Gastrointest Liver Physiol.

[22] Varum FJO, Veiga F, Sousa JS, Basit AW (2010). An investigation into the role of mucus thickness on mucoadhesion in the gastrointestinal tract of pig. Eur J Pharm Sci.

[23] Birchenough GMH, Johansson ME, Gustafsson JK, Bergström JH, Hansson GC (2015). New developments in goblet cell mucus secretion and function. Mucosal Immunol.

[24] Bron PA, Van Baarlen P, Kleerebezem M (2012). Emerging molecular insights into the interaction between probiotics and the host intestinal mucosa. Nat Rev Microbiol.

[25] Johansson ME, Hansson GC (2016). Immunological aspects of intestinal mucus and mucins. Nat Rev Immunol.

[26] Grondin JA, Kwon YH, Far PM, Haq S, Khan WI (2020). Mucins in intestinal mucosal defense and inflammation: learning from clinical and experimental studies. Front Immunol.

[27] Liu Y, Yu X, Zhao J, Zhang H, Zhai Q, Chen W (2020). The role of MUC2 mucin in intestinal homeostasis and the impact of dietary components on MUC2 expression. Int J Biol Macromol.

[28] Hasnain SZ, Evans CM, Roy M, Gallagher AL, Kindrachuk KN, Barron L, Dickey BF, Wilson MS, Wynn TA, Grencis RK, Thornton DJ (2011). Muc5ac: a critical component mediating the rejection of enteric nematodes. J Exp Med.

[29] Reid CJ, Harris A (1999). Expression of the MUC 6 mucin gene in development of the human kidney and male genital ducts. J Histochem Cytochem.

[30] Walsh MD, Clendenning M, Williamson E, Pearson S-A, Walters RJ, Nagler B, Packenas D, Win AK, Hopper JL, Jenkins MA (2013). Expression of MUC2, MUC5AC, MUC5B, and MUC6 mucins in colorectal cancers and their association with the CpG island methylator phenotype. Mod Pathol.

[31] Cerullo AR, Lai TY, Allam B, Baer A, Barnes WJP, Barrientos Z, Deheyn DD, Fudge DS, Gould J, Harrington MJ (2020). Comparative animal mucomics: inspiration for functional materials from ubiquitous and understudied biopolymers. ACS Biomater Sci Eng.

[32] Johansson ME, Sjövall H, Hansson GC (2013). The gastrointestinal mucus system in health and disease. Nat Rev Gastroenterol Hepatol.

[33] Luan Y, Xu W (2007). The structure and main functions of aminopeptidase N. Curr Med Chem.

[34] Wickström M, Larsson R, Nygren P, Gullbo J (2011). Aminopeptidase N (CD13) as a target for cancer chemotherapy. Cancer Sci.

[35] Stoian A, Rowland RRR, Petrovan V, Sheahan M, Samuel MS, Whitworth KM, Wells KD, Zhang J, Beaton B, Cigan M, Prather RS (2020). The use of cells from ANPEP knockout pigs to evaluate the role of aminopeptidase N (APN) as a receptor for porcine deltacoronavirus (PDCoV). Virology.

[36] Chen L, Lin Y-L, Peng G, Li F (2012). Structural basis for multifunctional roles of mammalian aminopeptidase N. Proc Natl Acad Sci USA.

[37] Yeager CL, Ashmun RA, Williams RK, Cardellichio CB, Shapiro LH, Look AT, Holmes KV (1992). Human aminopeptidase N is a receptor for human coronavirus 229E. Nature.

[38] Delmas B, Gelfi J, L'Haridon R, Sjöström H, Laude H (1992). Aminopeptidase N is a major receptor for the enteropathogenic coronavirus TGEV. Nature.

[39] Li B, Ge J, Li Y (2007). Porcine aminopeptidase N is a functional receptor for the PEDV coronavirus. Virology.

[40] Delmas B, Gelfi J, Sjöström H, Noren O, Laude H (1994). Further characterization of aminopeptidase-N as a receptor for coronaviruses. coronaviruses.

[41] Sun X, Li L, Pan L, Wang Z, Chen H, Shao C, Yu J, Ren Y, Wang X, Huang X (2021). Infectious bronchitis virus: identification of *Gallus gallus* APN high-affinity ligands with antiviral effects. Antivir Res.

[42] Luo L, Wang S, Zhu L, Fan B, Liu T, Wang L, Zhao P, Dang Y, Sun P, Chen J (2019). Aminopeptidase N-null neonatal piglets are protected from transmissible gastroenteritis virus but not porcine epidemic diarrhea virus. Sci Rep.

[43] Li W, Luo R, He Q, van Kuppeveld FJ, Rottier PJ, Bosch B-J (2017). Aminopeptidase N is not required for porcine epidemic diarrhea virus cell entry. Virus Res.

[44] Cui T, Theuns S, Xie J, Broeck WVd, Nauwynck HJ (2020). Role of porcine aminopeptidase N and sialic acids in porcine coronavirus infections in primary porcine enterocytes. Viruses.

[45] Matteucci E, Giampietro O (2009). Dipeptidyl peptidase-4 (CD26): knowing the function before inhibiting the enzyme. Curr Med Chem.

[46] Boonacker E, Van Noorden CJ (2003). The multifunctional or moonlighting protein CD26/DPPIV. Eur J Cell Biol.

[47] Raj VS, Mou H, Smits SL, Dekkers DHW, Müller MA, Dijkman R, Muth D, Demmers JAA, Zaki A, Fouchier RAM, Thiel V, Drosten C, Rottier PJM, Osterhaus ADME, Bosch BJ, Haagmans BL (2013). Dipeptidyl peptidase 4 is a functional receptor for the emerging human coronavirus-EMC. Nature.

[48] Cameron K, Rozano L, Falasca M, Mancera RL (2021). Does the SARS-CoV-2 spike protein receptor binding domain interact effectively with the DPP4 (CD26) receptor? A molecular docking study. Int J Mol Sci.

[49] Li Y, Zhang Z, Yang L, Lian X, Xie Y, Li S, Xin S, Cao P, Lu J (2020). The MERS-CoV receptor DPP4 as a candidate binding target of the SARS-CoV-2 spike. iScience.

[50] Yamamoto K, Takeshita H, Rakugi H (2020). ACE2, angiotensin 1–7 and skeletal muscle: review in the era of COVID-19. Clin Sci.

[51] Hoffmann M, Kleine-Weber H, Schroeder S, Krüger N, Herrler T, Erichsen S, Schiergens TS, Herrler G, Wu N-H, Nitsche A (2020). SARS-CoV-2 cell entry depends on ACE2 and TMPRSS2 and is blocked by a clinically proven protease inhibitor. Cell.

[52] Li W, Moore MJ, Vasilieva N, Sui J, Wong SK, Berne MA, Somasundaran M, Sullivan JL, Luzuriaga K, Greenough TC (2003). Angiotensin-converting enzyme 2 is a functional receptor for the SARS coronavirus. Nature.

[53] Paoloni-Giacobino A, Chen H, Peitsch MC, Rossier C, Antonarakis SE (1997). Cloning of the TMPRSS2 gene, which encodes a novel serine protease with transmembrane, LDLRA, and SRCR domains and maps to 21q22.3. Genomics.

[54] Bertram S, Heurich A, Lavender H, Gierer S, Danisch S, Perin P, Lucas JM, Nelson PS, Pöhlmann S, Soilleux EJ (2012). Influenza and SARS-coronavirus activating proteases TMPRSS2 and HAT are expressed at multiple sites in human respiratory and gastrointestinal tracts. PLoS ONE.

[55] Glowacka I, Bertram S, Müller MA, Allen P, Soilleux E, Pfefferle S, Steffen I, Tsegaye TS, He Y, Gnirss K (2011). Evidence that TMPRSS2 activates the SARS-coronavirus spike-protein for membrane fusion and reduces viral control by the humoral immune response. J Virol.

[56] Sserwadda M, Nevejan N, Ntanzi R, Cornillie P, Van den Broeck W, Van Stappen G (2020). Ontogenetic development of the gastrointestinal tract of African lungfish larvae *Protopterus aethiopicus* (Heckel 1851): a light microscopy study. Aquac Res.

[57] Liu J, Wang H-W, Lin L, Miao C-Y, Zhang Y, Zhou B-H (2019). Intestinal barrier damage involved in intestinal microflora changes in fluoride-induced mice. Chemosphere.

[58] Ortiz ME, Thurman A, Pezzulo AA, Leidinger MR, Klesney-Tait JA, Karp PH, Tan P, Wohlford-Lenane C, McCray PB, Meyerholz DK (2020). Heterogeneous expression of the SARS-Coronavirus-2 receptor ACE2 in the human respiratory tract. EBioMedicine.

[59] Mencucci R, Favuzza E, Becatti M, Tani A, Mazzantini C, Vignapiano R, Fiorillo C, Pellegrini-Giampietro D, Manetti M, Marini M (2021). Co-expression of the SARS-CoV-2 entry receptors ACE2 and TMPRSS2 in healthy human conjunctiva. Exp Eye Res.

[60] Chattha KS, Roth JA, Saif LJ (2015). Strategies for design and application of enteric viral vaccines. Annu Rev Anim Biosci.

[61] van Wettere WHEJ, Willson NL, Pain SJ, Forder REA (2016). Effect of oral polyamine supplementation pre-weaning on piglet growth and intestinal characteristics. Animal.

[62] Hedemann MS, Højsgaard S, Jensen BB (2003). Small intestinal morphology and activity of intestinal peptidases in piglets around weaning. J Anim Physiol Anim Nutr (Berl).

[63] Wang H, Yang L, Qu H, Feng H, Wu S, Bao W (2019). Global mapping of H3K4 trimethylation (H3K4me3) and transcriptome analysis reveal genes involved in the response to epidemic diarrhea virus infections in pigs. Animals.

[64] Overeem AW, Posovszky C, Rings EH, Giepmans BN, van IJzendoorn SC (2016). The role of enterocyte defects in the pathogenesis of congenital diarrheal disorders. Dis Model Mech.

[65] Schneeberger K, Roth S, Nieuwenhuis EES, Middendorp S (2018). Intestinal epithelial cell polarity defects in disease: lessons from microvillus inclusion disease. Dis Model Mech.

[66] Groschwitz KR, Hogan SP (2009). Intestinal barrier function: molecular regulation and disease pathogenesis. J Allergy Clin Immunol.

[67] Luo X, Guo L, Zhang J, Xu Y, Gu W, Feng L, Wang Y (2017). Tight junction protein occludin is a porcine epidemic diarrhea virus entry factor. J Virol.

[68] Tang X, Liu H, Yang S, Li Z, Zhong J, Fang R (2016). Epidermal growth factor and intestinal barrier function. Mediat Inflamm.

[69] Gil RS, Vagnarelli P (2018). Ki-67: more hidden behind a ‘classic proliferation marker’. Trends Biochem Sci.

[70] Zenewicz LA, Flavell RA (2011). Recent advances in IL-22 biology. Int Immunol.

[71] Gourbeyre P, Berri M, Lippi Y, Meurens F, Vincent-Naulleau S, Laffitte J, Rogel-Gaillard C, Pinton P, Oswald IP (2015). Pattern recognition receptors in the gut: analysis of their expression along the intestinal tract and the crypt/villus axis. Physiol Rep.

[72] Everaert N, Van Cruchten S, Weström B, Bailey M, Van Ginneken C, Thymann T, Pieper R (2017). A review on early gut maturation and colonization in pigs, including biological and dietary factors affecting gut homeostasis. Anim Feed Sci Technol.

[73] Karam SM (1999). Lineage commitment and maturation of epithelial cells in the gut. Front Biosci.

[74] Röhe I, Hüttner FJ, Plendl J, Drewes B, Zentek J (2018). Comparison of different histological protocols for the preservation and quantification of the intestinal mucus layer in pigs. Eur J Histochem.

[75] Yang X, Forier K, Steukers L, Van Vlierberghe S, Dubruel P, Braeckmans K, Glorieux S, Nauwynck HJ (2012). Immobilization of pseudorabies virus in porcine tracheal respiratory mucus revealed by single particle tracking. PLoS ONE.

[76] Whitworth KM, Rowland RR, Petrovan V, Sheahan M, Cino-Ozuna AG, Fang Y, Hesse R, Mileham A, Samuel MS, Wells KD (2019). Resistance to coronavirus infection in amino peptidase N-deficient pigs. Transgenic Res.

[77] Shirato K, Maejima M, Islam MT, Miyazaki A, Kawase M, Matsuyama S, Taguchi F (2016). Porcine aminopeptidase N is not a cellular receptor of porcine epidemic diarrhea virus, but promotes its infectivity via aminopeptidase activity. J Gen Virol.

[78] Nelli RK, Roth JA, Gimenez-Lirola LG (2022). Distribution of coronavirus receptors in the swine respiratory and intestinal tract. Vet Sci.

[79] Krejner-Bienias A, Grzela K, Grzela T (2021). DPP4 inhibitors and COVID-19–holy grail or another dead end?. Arch Immunol Ther Exp.

